# A new electrochemical impedance biosensor based on aromatic thiol for alpha-1 antitrypsin determination

**DOI:** 10.3906/kim-2007-6

**Published:** 2021-02-17

**Authors:** Hülya YAĞAR, Hakkı Mevlüt ÖZCAN, Osman MEHMET

**Affiliations:** 1 Department of Chemistry, Faculty of Science, Trakya University, Edirne Turkey

**Keywords:** Alpha-1 antitrypsin, electrochemical immunosensor, 4-mercaptophenylacetic acid, pulmonary emphysema, Alzheimer’s disease

## Abstract

Alpha-1 antitrypsin (A1AT) is one of the acute phase proteins which are synthesized in the liver. A1AT inhibits the activity of many proteases, but its main task is to protect the lungs from the attack of neutrophil elastase. In an autosomal hereditary disease known as alpha-1 antitrypsin deficiency, the A1AT level in blood serum decreases, increasing the risk of developing emphysema, liver apoptosis, and liver cancer. Thus, the detection of A1AT concentration in blood serum is very important. In this study, an impedimetric biosensor was developed, forming an SAM (self-assembled monolayer) with 4-mercaptophenylacetic acid (4MPA) on the surface of the gold electrode. An A1AT biosensor was constructed using immobilization of an A1AT-specific antibody (anti-A1AT) after activating the carboxyl groups of 4MPA with EDC/NHS. Each immobilization stage was characterized by using electrochemical impedance spectroscopy, cyclic voltammetry, and scanning electron microscopy with energy dispersive X-ray spectroscopy. With the designed biosensor, precise and highly reproducible results were obtained for A1AT concentrations in the range of 100–600 µg/mL. A1AT detection was also successfully carried out in artificial serum solutions spiked with A1AT.

## 1. Introduction

Acute phase response occurs after many homeostatic mechanisms, tissue damage, and infections under normal conditions in vertebrates. Plasma proteins synthesized during the acute phase response are called acute phase proteins (AFPs); they initiate physiological changes [1]. The quantitative analysis of AFPs may be critically important for the diagnosis and treatment of some diseases which are directly associated with the level of relevant AFPs in serum. Alpha-1 antitrypsin (A1AT) is an AFP; it is a single-chain glycoprotein synthesized in the liver. It consists of 394 amino acids and weighs 52 kDa. It is a member of the class of protease inhibitor proteins known as serine protease inhibitors (serpins) [2]. A1AT is the most abundant serpin in plasma and is the major inhibitor of serine proteases present in circulation and tissue [3]. Around 34 mg of A1AT is synthesized per kg of body weight in a day [4]. 

A1AT protects tissues, especially lungs, from protease enzymes, especially neutrophil elastase, by using the inhibition mechanism. Neutrophil elastase has a high potential to destroy the matrix components of the lungs. It is responsible for breaking down the elastin proteins and other components of the extracellular matrix which provides flexibility to the lungs during air exchange [5]. In addition to inhibition of neutrophil elastase, A1AT also inhibits other human serine proteases including trypsin, chymotrypsin G, plasmin, thrombin, factor X, and plasminogen [2]. 

The level of A1AT normally present in serum is 80–220 mg/dL. The normal concentration range changes from 20 to 48 µM when the nephelometry method is used; the range is 150 mg/dL to 350 mg/dL when the radial immunodiffusion method is used [6]. This concentration typically increases manifold in acute inflammation or postinfection, as well as in a number of conditions ranging from acute community-acquired pneumonia to postsurgery [7]. However, a decrease in A1AT level in blood is clinically more important, as it indicates a disorder called alpha-1 antitrypsin deficiency (AATD) caused by mutations in the gene encoding A1AT protein. A decrease in A1AT production below 30% of normal levels creates significant risk for lung diseases in early adulthood. An A1AT concentration in serum lower than 50 mg/dL is associated with a high risk of developing emphysema; there is a slightly increased risk at A1AT concentrations of 50–80 mg/dL [8]. Inadequate amounts of A1AT in blood cause destruction of the lung tissue, especially alveolar walls, by neutrophil elastase. If the structure of the lungs deteriorates, they become unable to function, and various diseases such as emphysema and chronic obstructive pulmonary disease (COPD) develop. Liver damage and jaundice have been observed in about 10% of newborns with AATD. When A1AT synthesized in the liver is not secreted into blood, A1AT that accumulates in hepatocyte cells may cause liver cirrhosis and subsequent liver cancer [9]. Moreover, in recent years, A1AT has been suggested as a novel biomarker for the very early stages of liver cancer and Alzheimer’s disease [10,11]. 

There is no permanent cure for AATD with current technology. However, its diagnosis is vital because people diagnosed with AATD should lead the highest quality of life possible depending on the level of deficiency. Untreated A1AT deficiency can lead to a progressive and fatal result for the majority of affected people. It is important for people with A1AT deficiency and a clinical diagnosis of emphysema to receive parenteral A1AT enhancement therapy [8], so that they can survive much longer and lead a quality life. The symptoms of A1AT deficiency are often confused with those of COPD [12]. Therefore, according to a statement made by the World Health Organization (WHO) based on the data available in 1997, it is suggested that people suffering from COPD and young-adult individuals diagnosed with asthma should undergo a quantitative A1AT test at least once [13].

Today, the immune nephelometric method is used commonly for A1AT quantitative analysis [14]. Protein electrophoresis and immunofixation electrophoresis are also applied to identify abnormal bands seen on A1AT, while isoelectrofocusing electrophoresis is used for detecting A1AT deficiency variants1Lab test online UK (2020). Alpha-1 Antitrypsin [online]. Website https://labtestsonline.org.uk/tests/alpha-1-antitrypsin [accessed 23 October 2020]. . Although these tests are sufficient to make the diagnosis, they are not used in every health institution due to some disadvantages such as the long preparation steps, the necessity that they be performed by experts, the difficulty of the analysis method, and the high cost of the equipment. Therefore, it is important to develop simple and specific methods for the diagnosis of AATD. 

The aim of this study is to develop a specific, cost-effective, and easily applicable biosensor system to detect the amount of A1AT protein in serum. In the literature, there are two biosensor studies related to the detection of A1AT. One of them is the sandwich-type antibody–aptamer biosensor prepared using A1AT aptamer, which detects A1AT concentration using the surface plasmon resonance (SPR) method [10]. Another sandwich-type biosensor is composed of alkaline phosphatase-labeled A1AT antibody functionalized silver nanoparticles (ALP-A1AT Ab-Ag NPs) and 3,4,9,10-perylene tetracarboxylic acid/carbon nanotubes (PTCA-CNTs), which detects the electrochemical response of 4-aminophenol (AP) produced enzymatically from 4-amino phenyl phosphate (APP) with differential pulse voltammetry (DPV) [15].

The A1AT biosensor developed based on immune recognition of the A1AT antibody (anti-A1AT) in the present study is a label-free biosensor. Label-free electrochemical immunosensors provide a lot of beneﬁts such as ease of use, fast response, low cost, and the ability to directly detect changes in the signals originated from the specific antibody–antigen interaction without the need for any labels [16]. Since label-free strategies reduce the number of immobilization stages, the fabrication protocols of label-free electrochemical immunosensors are simple and low-cost. The electrode fabrication procedure also has an important role in the success of the biosensor [17]. The proposed A1AT biosensor in the present study was prepared using only a few chemicals. When this biosensor was being constructed, a self-assembled monolayer (SAM) was created with 4-mercaptophenylacetic acid (4MPA) on a gold electrode; anti-A1AT molecules were then immobilized to the end carboxyl groups of 4MPA. The forming of SAM is a surface modification method frequently preferred during fabrication of a novel biosensor due to its attractive characteristics including simplicity, stability, and biocompatibility. To form SAM using aliphatic thiols to modify the gold electrode surface in the biosensor design is often mentioned in the literature [18–22]; however, the use of aromatic thiols in biosensor studies is much less common [23–25]. 

In this study, the aromatic thiol 4MPA was used for the first time to form SAM in the fabrication of an electrochemical-based impedimetric immunosensor for detection of A1AT protein.

## 2. Materials and methods

### 2.1. Materials

Alpha-1 antitrypsin (A1AT), anti-alpha-1 antitrypsin antibody (anti-A1AT), 4-mercaptophenylacetic acid (4MPA), 1-ethyl-3-(3-dimetilaminopropil) carbodiimide (EDC), N-hydroxysuccinimide (NHS), serum replacement solution 3, potassium ferricyanide, and potassium ferrocyanide were purchased from Sigma-Aldrich (St. Louis, MO, USA). The A1AT and anti-A1AT were diluted with ultrapure water at certain concentrations and stored at –20 °C until use. The artificial serum solution was prepared by using 4.5 mM KCl, 5 mM CaCl_2_, 4.7 mM (D+)‐glucose, 2.5 mM urea, and 145 mM NaCl in the serum replacement solution 3, which contained only human proteins such as human serum albumin, human transferrin, and human recombinant insulin. The Ag/AgCl reference electrode, platinum counter electrode, and gold working electrode were provided by BASi (Warwickshire, UK). The Ag/AgCl reference electrode was stored in 3 M KCl solution until use. All electrochemical experiments were carried out using a Gamry Interface 1000 Potentiostat/Galvanostat (Gamry Instruments, Warminster, PA, USA) interfaced with a PC equipped with ECM Analyst software (Gamry Instruments).

### 2.2. Preparation of the biosensor

Before starting the biosensor preparation, electrode cleaning is an essential and important step for removing impurities on the electrode surface that might remain from previous experiments. For this purpose, aluminum oxide (Al_2_O_3_ <50 nm partial size) was mixed with ultrapure water on synthetic rayon (Buehler Microcloth PSA, Buehler, Coventry, UK); the surface of the gold electrode was rubbed gently with aluminum oxide paste. In order to remove the aluminum oxide particles remaining on the electrode surface, the gold electrode was washed with ultrapure water, after which it was incubated in an ultrasonic bath sonicator for 5 min with both absolute ethanol (99.9%) (v/v) and ultrapure water. It was then dried with pure argon and referred to as a bare working electrode (Au-BARE).

The cleaned electrode was incubated for 16 h in 5 mM 4MPA solution prepared in ethanol (99.9%) (v/v) to form SAM on the surface of the Au-BARE electrode. It was then washed with ethanol (99.9%) (v/v) and ultrapure water to remove 4MPA molecules remaining on the electrode surface (Au-4MPA). In the second step, the Au–4MPA electrode was incubated with 0.2M/0.05M EDC/NHS (1:1) solution for 30 min to activate the carboxylic acid functional groups of 4MPA molecules. After the EDC/NHS activation step, the modified electrode was referred to as Au–4MPA–EDCNHS. In the last step, the Au–4MPA–EDCNHS electrode was incubated with 200 µg/mL anti-A1AT for 1 h. Finally, the biosensor was washed with ultrapure water to remove unbound antibody molecules on the electrode surface, and dried with pure argon. The modified electrode is referred to as Au–4MPA–EDCNHS–anti-A1AT. All steps performed for the construction of the biosensor are illustrated in Scheme. 

**Scheme:**
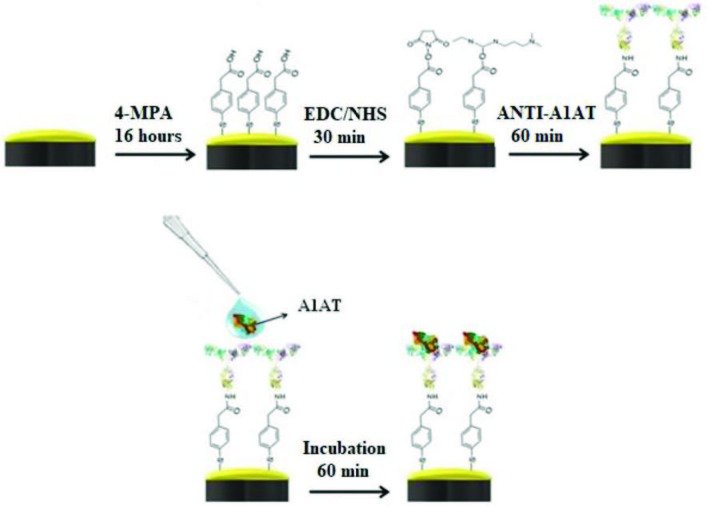
Construction of the biosensor.

### 2.3. Principals of the electrochemical measurements 

The A1AT immunosensor proposed in the present study is based on electrochemical impedance spectroscopy (EIS). Electrochemical immunosensors have attracted a lot of attention in the area of biosensor construction in recent years. EIS is frequently used to improve label-free and selective biosensors. This technique is used to evaluate the specific interactions between antigens and antibodies on the electrode surfaces by measuring their capacitance or interfacial charge–transfer resistance. It has some advantages, including simple application procedure, low cost, fast detection, and ease of miniaturization [16,17,26]. 

**Figure 1:**
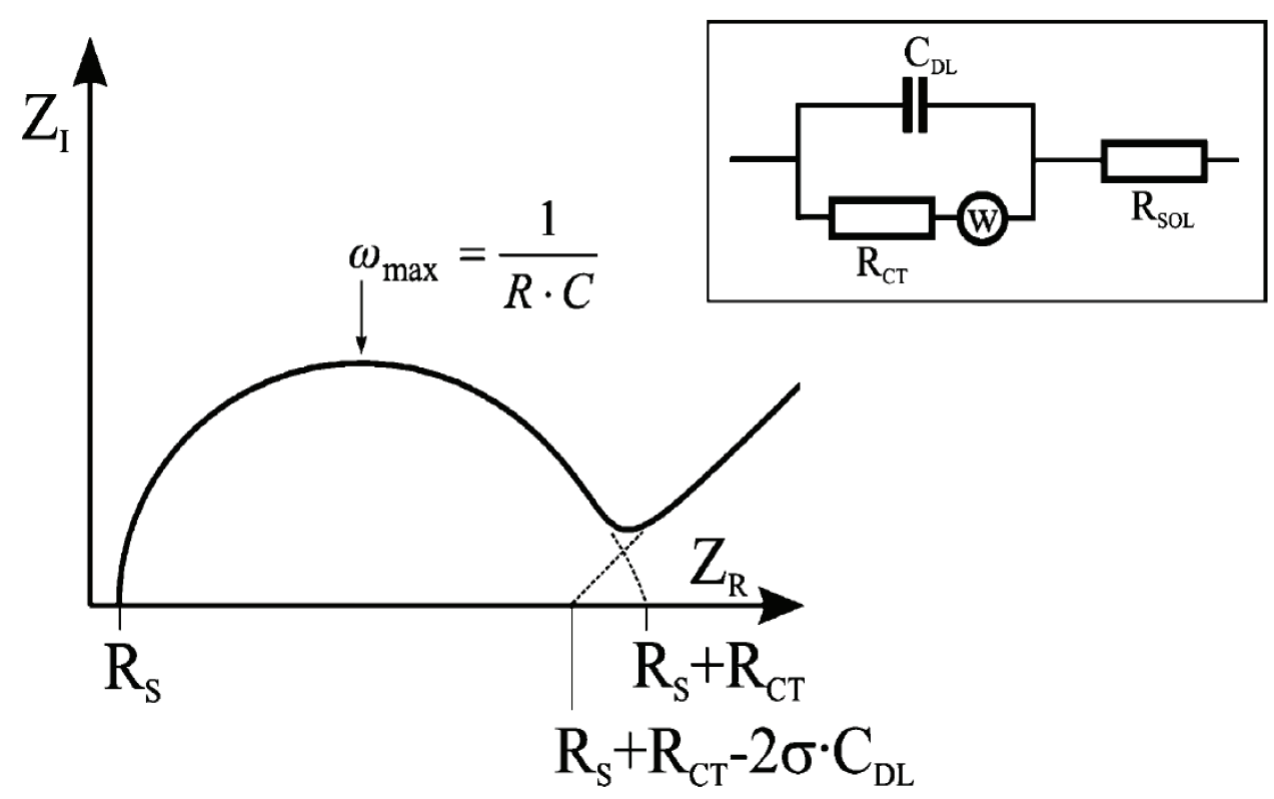
Randles equivalent circuit model for electrode in contact with an electrolyte.

In this study, EIS was used to check the success of the immobilization process at each step of immobilization of the A1AT antibody on the gold electrode surface, and then to determine the A1AT amount that was bound to antibody molecules immobilized on the biosensor surface. Impedance can be defined as the total resistance of the system at a fixed potential. Equivalent circuit models are used to determine experimental impedance data by using impedance elements arranged in serial and/or in parallel. The Randles equivalent circuit model is the most frequently used equivalent circuit model for this purpose. Most electrochemical systems are analyzed according to this procedure. Where the Randles circuit is defined as a system in which an electrolyte and an electrode are in contact, the solution resistance is Rs, the charge transfer resistance is Rct, the double layer capacitance is C_DL_, and Warburg impedance is W. Rs and Rct values can be easily determined in the Nyquist graph shown in Figure 1. 

In this study, Gamry Potentiostat ECM Analyst software was used to determine the Rct values. The calibration graph was plotted as calculated Rct values versus different concentrations of A1AT. 

Rct values were calculated using the following equation:

∆R_ct_ = R_ct(Au–4MPA–EDCNHS–anti-A1AT-A1AT)_ – R_ct(Au–4MPA–EDCNHS–anti-A1AT)_,

where R_ct(Au-4MPA-EDCNHS-Anti-A1AT)_ was defined as Rct determined after anti-A1AT was immobilized on the gold electrode surface, and R_ct(Au-4MPA-EDCNHS-Anti-A1AT-A1AT)_ was defined as the Rct after A1AT was attached to anti-A1AT. (K_3_[Fe(CN)_6_/ K_4_[Fe(CN)_6_] solution (1:1; v/v) was used as the redox probe solution for all EIS and CV measurements. Cyclic voltammograms were obtained in the potential range of –0.5 to 1.0 V with a step size of 1 mV and a scanning rate of 50 mV/s. EIS spectrums were obtained at 5 mV alternating current; impedance spectra frequency range was 0.05–10,000 Hz.

### 2.4. Morphological characterization of biosensor

Structural observations of the electrode surface after each immobilization step of the A1AT biosensor were performed with a field-emission scanning electron microscope (SEM) (EVO/LS10 ZEISS) at the Technology Research and Development Centre of Trakya University. An acceleration voltage of 5 kV was used to acquire SEM images.

## 3. Results and discussion

### 3.1. Anti-A1AT immobilization

A1AT immobilization was performed as described in Scheme. EIS spectra and the cyclic voltammograms obtained for each immobilization step of the anti-A1AT are shown in Figures 2a and 2b. Rct values calculated using Gamry ECM Analyst software for each immobilization step of the anti-A1AT are given in Table 1.

**Figure 2a:**
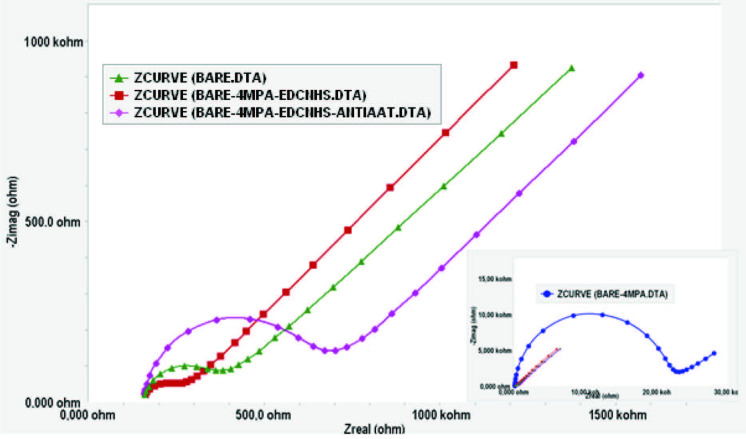
EIS spectra of each immobilization step of the Anti-A1AT [-▲-▲- (green): bare electrode, -●-●- (blue): 4MPA, -■-■- (orange): 4MPA-EDCNHS, -♦-♦- (pink): 4MPA-EDCNHS-ANTIA1AT].

**Figure 2b:**
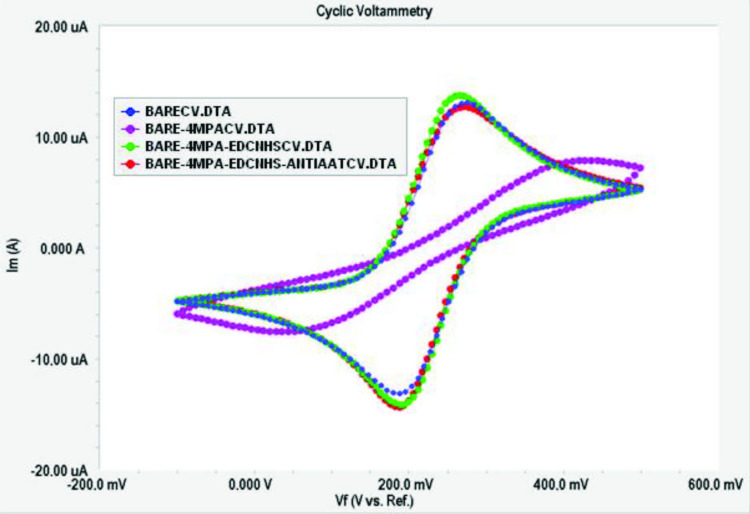
Cyclic voltammograms of each immobilization step of the Anti-A1AT [-●-●- (blue): bare electrode, -●-●- (pink): 4MPA, -●-●- (green): 4MPA-EDCNHS, -●-●- (orange): 4MPA-EDCNHS-ANTIA1AT] .

**Table 1:** Rct of each immobilization step of the Anti-A1AT.

Electrode	Rct (Ω)	Standard deviation (±)
Au-Bare	162.9	26.11
Au-4MPA	31000	4008.00
Au-4MPA-EDCNHS	126.4	16.18
Au-4MPA-EDCNHS-ANTIA1AT	399.5	31.20

As shown in Table 1 and Figure 2a, after the bare electrode was modified using 4MPA, the Rct values greatly increased. This is an expected result since the carboxyl groups of 4MPA probably prevented the negative charge from reaching the electrode surface. 

As shown in Figure 2a, the Rct value obtained for the Au–MPA electrode decreased significantly after being activated with EDC/NHS. This decrease indicates that the activation of the carboxyl groups by EDC was successful. EDC/NHS chemistry is a fairly well-known activation reaction. EDC is a zero-length crosslinking agent used to bind carboxyl groups to primary amines. The efficiency of EDC increases in the presence of NHS, which is a reagent used to activate carboxylic acid groups. Activated acids react with amines to form amides. In the last step, it was determined that the Rct value of the Au–4MPA–EDCNHS–anti-A1AT electrode increases when compared to that of the Au–4MPA–EDCNHS electrode. This increase was a result of covalent binding of the antibody to the EDC/NHS-activated carboxyl ends. It was an expected result that the antibody protein bound to the electrode surface would increase Rct. This result was confirmed by the change of anodic and cathodic currents observed in cyclic voltammograms (Figure 2b). 

CV was used to investigate the changes in the peak currents of the redox probe. The peak currents in CV depend on the surface composition of the gold electrode [27]. As shown in Figure 2b, the characteristic anodic and cathodic currents of the redox process were obtained for the Au-BARE electrode. After the bare electrode was modified with 4MPA, the peak currents changed significantly due to SAM formation. This meant that the negatively charged carboxyl groups of 4MPA prevented the negative charge from reaching the electrode surface [28]. When the carboxyl ends of 4MPA were activated by EDC/NHS, a significant increase of peak currents was observed. When the carboxyl group is activated, the intermediate product formed is positively charged and attracts the redox probe toward the electrode surface. Thus, an increase of currents is observed in cyclic voltammograms, as the conversion of more electrolytes on the electrode surface will cause an increase in the measured current [29]. Immobilization of anti-A1AT on the modified gold electrodes increased the insulating property of the electrode surface and prevented diffusion of the redox probe. Therefore, an increase in Rct and a decrease in the CV peak were observed.

Some studies related to the formation of SAM by using aliphatic thiols with carboxylic acid are reported in the literature, although there have been few studies about aromatic thiols. Ahmad and Moore and Kim and Lee modified the gold electrode surface using an aliphatic thiol (11-mercaptoundecanoic acid) to form SAM in the biosensor designs [9,15,30]. Ozcan, who is in our working group, used 12-mercaptododecanoicacid monolayers for the construction of a parathyroid hormone biosensor [29]. Similar to the results of our study, the aliphatic thiols with carboxylic acid used in these studies seemed to cause significant increases in Rct values. 

Some aromatic and aliphatic thiols including mercaptosuccinic acid (MSA), 4-mercaptobenzoic acid (4-MBA), and 4MPA have been investigated in terms of their ability to form SAM in one study. A small difference was determined between cyclic voltammograms of aromatic and aliphatic thiols. A decrease in biosensor response was reported because electrons were transferred to the gold electrode surface through the redox probe solution and conjugated double bonds in the aromatic ring [31].

#### 3.1.1. The optimization of A1AT immobilization steps

The regular SAM formation step on the gold electrode surface affects the success of subsequent immobilization steps in a biosensor design. In this study, the aromatic thiol 4MPA which was chosen to form SAM was used successfully for the design of an impedimetric biosensor for the first time in the literature. 

**Figure 3:**
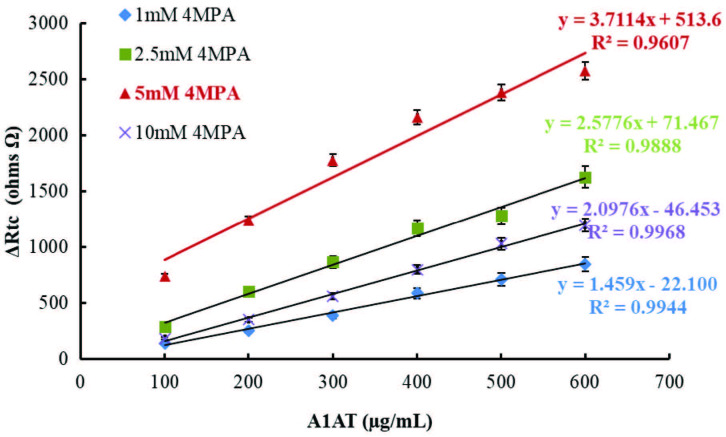
Calibration graphs of A1AT biosensors prepared using different 4-MPA concentration; [-♦-♦-(blue): 1.0 mM 4MPA, -■-■-(green): 2.5 mM 4MPA, -▲-▲-(red): 5.0 mM 4MPA, -X-X- (purple): 10.0 mM 4MPA].

The first step of the optimization of anti-A1AT immobilization conditions was determination of the effect of 4MPA concentration on SAM formation. For this purpose, four different biosensors were prepared by incubating the gold electrodes cleaned as described above with 1 mM, 2.5 mM, 5 mM, and 10 mM 4MPA solutions (in absolute ethanol 99.9%) for 16 h. The concentrations of all other chemicals used for the design of the biosensor were kept constant. EIS spectra at six different A1AT concentrations (100–600 µg/mL) were obtained for each prepared biosensor. Calibration graphs were plotted using A1AT concentrations versus Rct values calculated from EIS spectra, and are given in Figure 3.

As shown in Figure 3, the regression coefficient (R2) values were calculated as 0.9944, 0.9888, 0.9607, and 0.9968 from the calibration graphs plotted for the biosensors fabricated using 1 mM, 2.5 mM, 5 mM, and 10 mM 4MPA, respectively. Although the biosensor fabricated using 5 mM 4MPA had a relatively low R2 value, high biosensor responses were obtained. Therefore, the optimum concentration of anti-A1AT was selected as 5 mM. As depicted in Figure 3, the use of a low concentration of 4MPA (1 mM) caused the biosensor response to decrease. Since an insufficient SAM formation would cause insufficient antibody immobilization, a decrease in biosensor response was an expected result. In cases where aliphatic thiols are used for the formation of SAM, an increase in the biosensor response is normally expected with increased thiol concentration. However, the use of MPA at a high concentration of 10 mM decreased the response of the biosensor. The use of a very high aromatic thiol (10 mM) concentration for SAM formation may have caused irregular and unstable SAM formation due to the aromatic structure and volume of the molecule.

**Figure 4:**
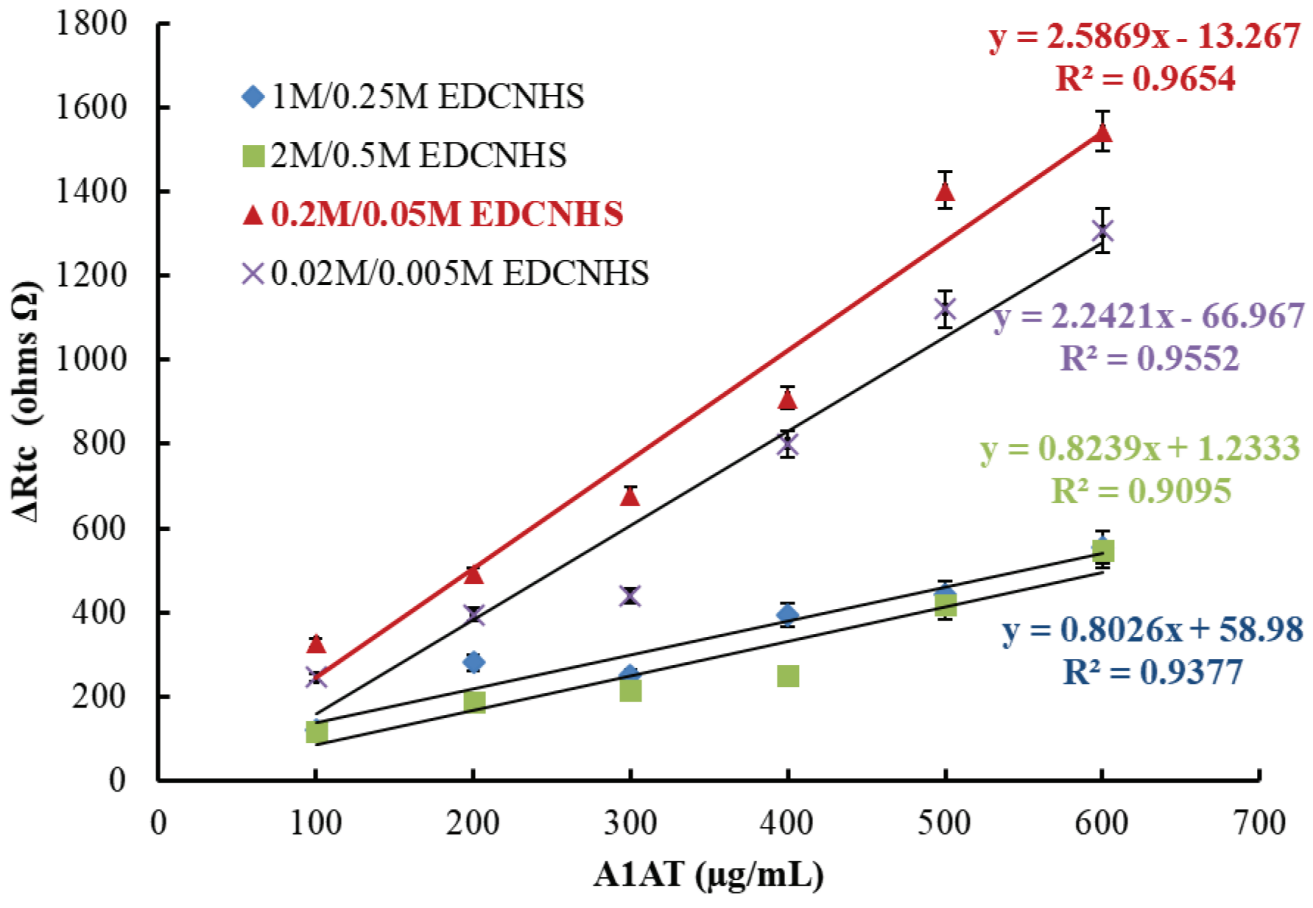
Effect of EDC/NHS concentration on biosensor response; [-♦-♦-(blue): 1.0 M/0.25 M EDC/NHS, -■-■-(green): 2.0 M/0.5 M EDC/NHS, -▲-▲-(red): 0.2 M/0.05 M EDC/NHS, -X-X- (purple): 0.02 M/0.005 M EDC/NHS].

In the second step, the optimization study for the concentration of the EDC/NHS couple was carried out to determine the amount of the EDC/NHS couple that activated the carboxyl ends of the 4MPA. For this purpose, 4 biosensors were fabricated using different concentrations of EDC/NHS solutions (0.02 M/0.005 M, 0.2 M/0.05 M, 2.0 M/0.5 M, and 1 M/2.5 M) by keeping the ratio of EDC to NHS constant. For better observation of the effect of EDC/NHS concentrations on immobilization, 4MPA and anti-A1AT concentrations were kept constant. Calibration graphs drawn between A1AT concentrations versus Rct values that were calculated from Nyquist curves of biosensors fabricated by using EDC/NHS solution mixtures prepared at the concentrations mentioned above are shown in Figure 4. R2 values obtained from the calibration graph plotted for the four biosensors fabricated by using 0.02 M/0.005 M, 0.2 M/0.05M, 1.0 M/0.25 M, and 2.0 M/0.5 M of the EDC/NHS pair were calculated as 0.9552, 0.9654, 0.9377, and 0.9095, respectively. In higher concentrations of the EDC/NHS pair, more anti-A1AT molecules are expected to be immobilized on the electrode surface. In contrast, as shown in Figure 4, decreases in biosensor responses were observed at high EDC/NHS concentrations. This may be because high EDC/NHS concentrations will create more mass on the SAM layers. Since more antibodies were immobilized on the electrode surface, high EDC/NHS concentrations may cause the collapse of the SAM layers.

In addition, the application of the mixtures of EDC/NHS of high concentrations (1.0 M/0.25M and 2.0 M/0.5 M) to the electrode surface was very difficult due to viscosity; adhesions were seen on the electrode surface after incubation for 30 min. Therefore, the concentrations of 0.2 M/0.05 M were selected as the optimum concentration of EDC/NHS by evaluating the slope, regression coefficient, and Rct values of the calibration curves.

**Figure 5:**
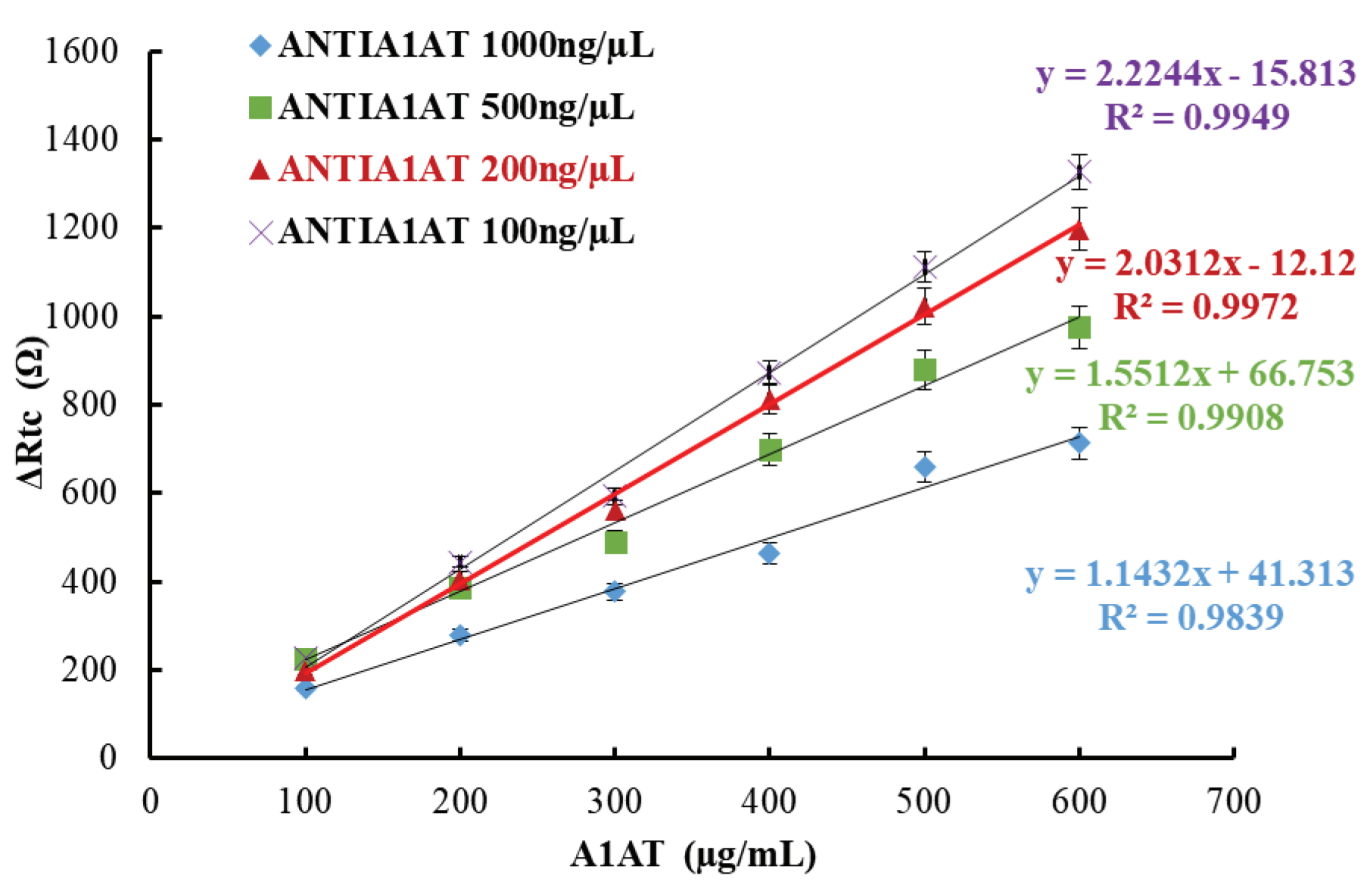
Effect of Anti-A1AT concentration on biosensor response; [-♦-♦-(blue): 1000 ng/μL Anti-A1AT, -■-■-(green): 500 ng/μL Anti-A1AT, -▲-▲-(red): 200 ng/μL Anti- A1AT, -X-X- (purple): 100 ng/μL Anti-A1AT].

In the final stage of the immobilization steps, four different biosensors were prepared using anti-A1AT solutions with concentrations of 100 µg/mL, 200 µg/mL, 500 µg/mL, and 1000 µg/mL to determine the effect of anti-A1AT concentration on the biosensor response. Calibration graphs drawn between the anti-A1AT concentrations and Rct values calculated from the Nyquist curves of the prepared biosensors are shown in Figure 5. As the amount of antibody to be immobilized increases, the charge transfer to the surface decreases and the Rct value of the Nyquist curve increases. However, binding more proteins to the surface increases the mass carried by the SAM layer, and consequently the load on the surface can cause deformations and collapses in the immobilization layers. In addition, as the amount of antibody increases, the bounding of the antibodies to the surface may be very tight; therefore, successful positioning and correct orientation of the antibody may not be possible. As shown in Figure 5, although the linearity coefficients are good for all biosensors, the biosensor response decreased when the A1AT concentration was increased. Comparing the linearity coefficients and Rct values obtained with biosensors fabricated using 100 µg/mL and 200 µg/mL anti-A1AT concentrations, it is appropriate to select both as optimum anti-A1AT concentrations. Although the responses of the biosensor fabricated using 100 µg/mL anti-A1AT were not significantly different from the responses of the biosensor fabricated using 200 µg/mL, 200 µg/mL was chosen as the optimum anti-A1AT concentration, as it was thought that the biosensor fabricated using 200 µg/mL Anti-A1AT would bind more A1AT proteins during measurement. 

The Laviron equation is useful for estimating the concentration of electroactive species on an electrode surface; it is also used to prove the success of immobilization [32,33]. The Laviron equation is given as Q = n × F × A × Г; where F is the constant of Faraday, n is the slope of the graphs plotted versus the peak currents (determined from cyclic voltammograms obtained at 10 different scan rates [10 mV s–1 to 100 mV s–1]), A is the surface area of electrode, г is the amount of electroactive molecule on the electrode surface, and Q is the charge [34]. 

The amounts of the electroactive molecules on the bare electrode and biosensor (Au–4MPA–EDCNHS–anti-A1AT) were calculated as 1.82 × 10–4 mol/cm2, and 2.01 × 10–4 mol/cm2, respectively. The value of the biosensor was higher than that of the obtained value with the bare electrode, indicating that the immobilization of anti-A1AT was successful. 

**Figure 6:**
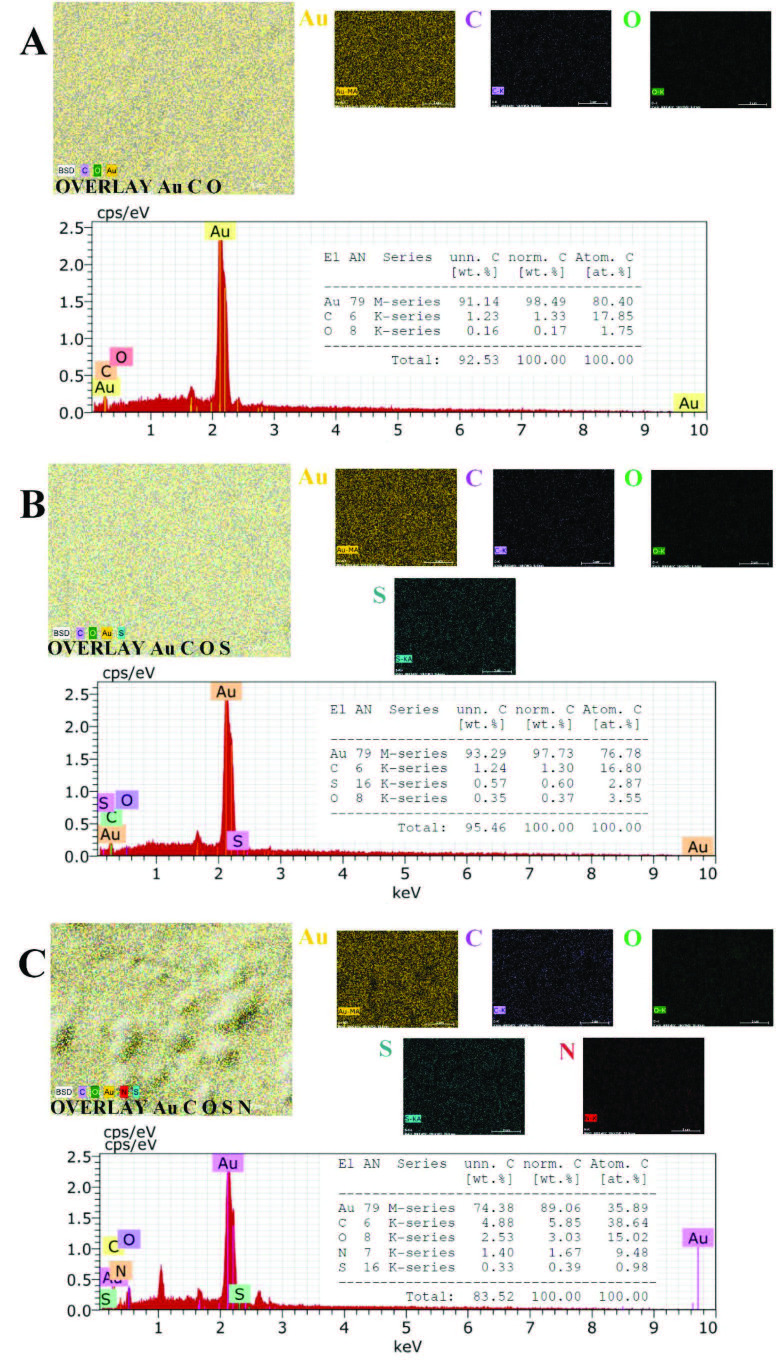
EDX elemental mapping and spectra of Au-BARE (A), Au-4MPA (B), Au-4MPA-EDCNHS-ANTI-A1AT (C).

**Figure 7:**
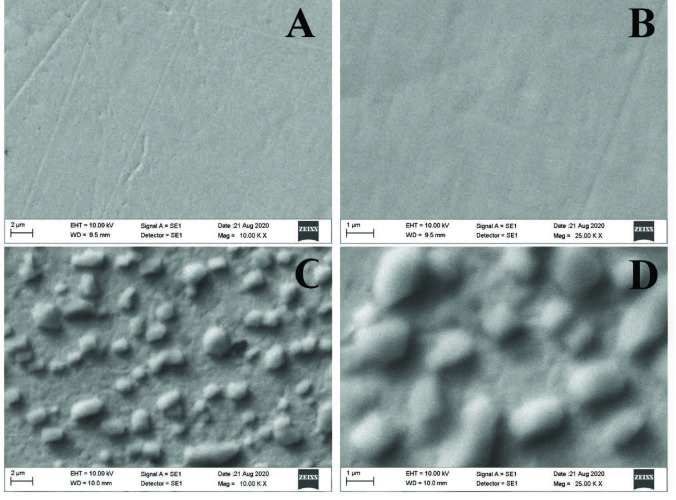
SEM images of immobilization steps of the proposed biosensor; Au-BARE (A), Au-4MPA (B), Au-4MPA-EDCNHS-ANTIA1AT (C, D).

We also used SEM with EDX to identify the fundamental composition of the electrode surface in each of the immobilization steps. EDX elemental mapping and spectra are given in Figures 6A–6C. After modification of the gold electrode surface with 4MPA, the element sulfur (S, light green) was observed differently from the Au bare electrode surface (Figure 6B). This result showed that the bare gold electrode surface was modified with a sulfur-containing element. Furthermore, as shown in Figure 6C, Au, O, C, S, and nitrogen (N, red) elements were observed on the electrode surface after anti-A1AT immobilization. Unlike the electrode surface modified with 4 MPA, the observation of the element N and the increase in the percentages of the elements C and O may be due to the antibody bound to the surface. The SEM images are shown in Figures 7A–7D. Figure 7A (smooth gold surface) shows Au bare electrode (Au–BARE), Figure 7B (cloudlike structure) shows the image after modification of 4MPA (Au–BARE–4MPA), and Figures 7C and 7D shows the images after anti-A1AT immobilization (Au–4MPA–EDCNHS–anti-A1AT). Figures 7C and 7D show that the immobilization of anti-A1AT created a sponge-like structure on the electrode surface. Thus, EDX images were also supported by SEM images.

### 3.2. Analytical characteristics of the biosensor

A1AT determination was carried out in the concentration range of 100–600 µg/mL with the biosensors fabricated in the optimum conditions mentioned above (4MPA [5 mM], EDC/NHS [0.2 M/0.05 M], anti-A1AT [200 µg/mL]). For this purpose, EIS and CV measurements were taken using prepared biosensors at six different A1AT concentrations, and Rct values were calculated for each concentration. EIS spectra and the cyclic voltammogram obtained as biosensor responses are shown in Figures 8a and 8b, respectively.

**Figure 8a:**
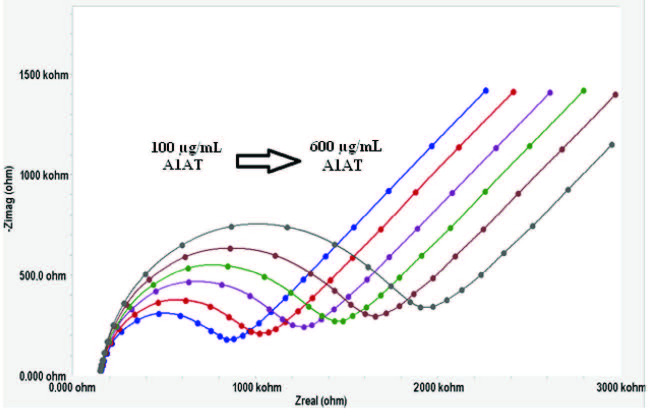
Electrochemical impedance spectra obtained as Nyquist curves for different A1AT concentrations.

**Figure 8b:**
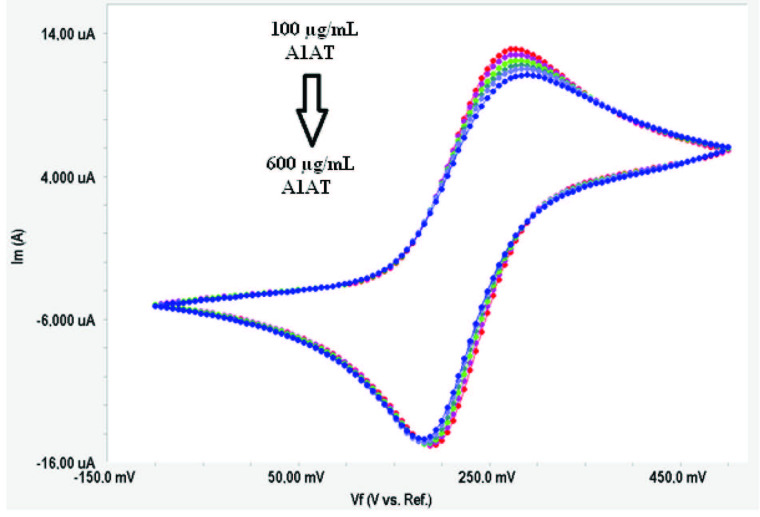
Cyclic voltammograms obtained for different A1AT concentrations.

The relationship between A1AT concentrations and Rct values is seen in the EIS spectra given in Figure 8a. As shown in Figure 8a, the increase in A1AT concentration caused the Rct values to increase. This linear relationship between A1AT concentrations and Rct values was also confirmed by the cyclic voltammograms, as shown in Figure 8b. 

**Figure 9:**
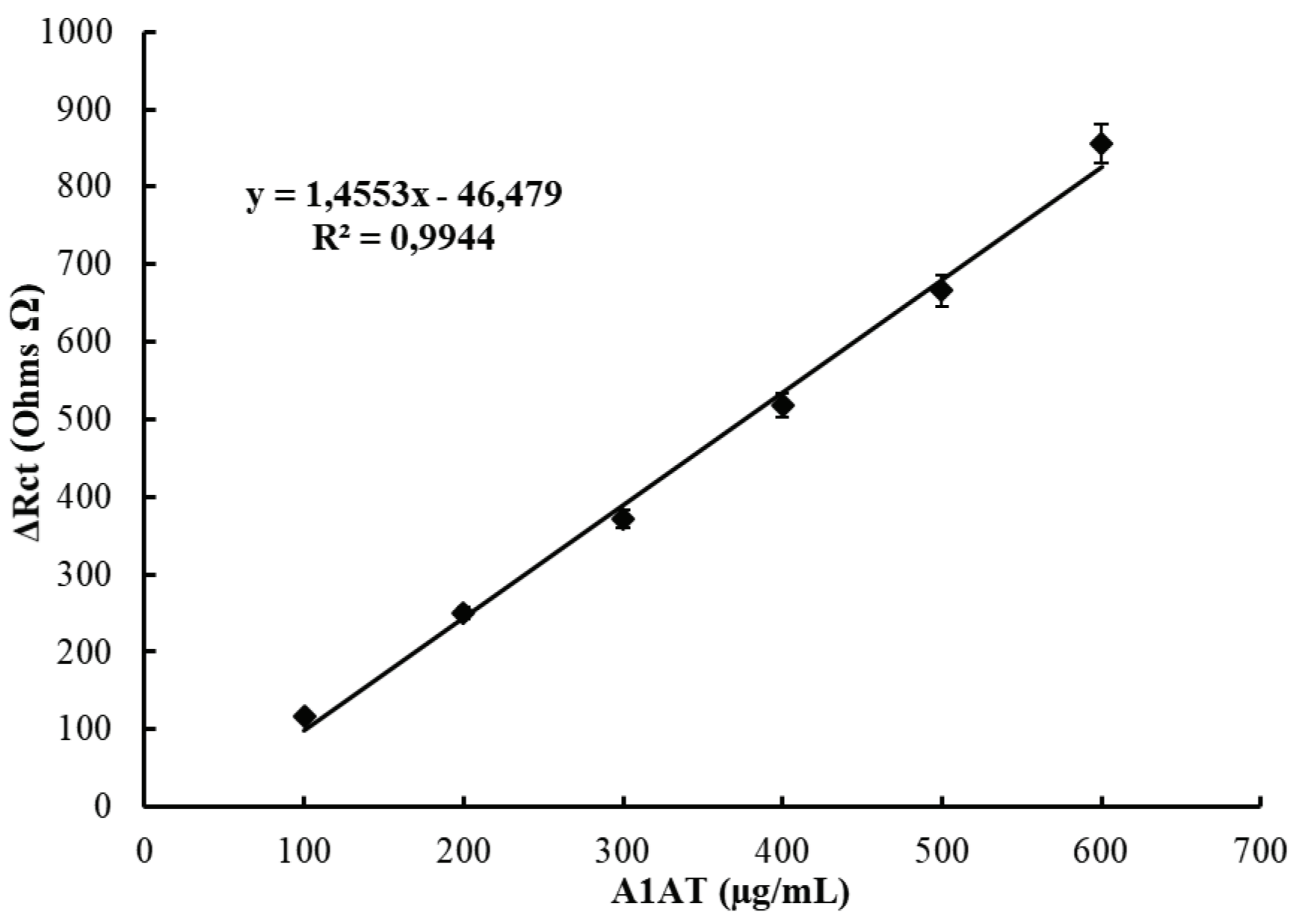
A1AT calibration graph based on the impedance changes (ohms) resulting from A1AT analysis with the proposed A1AT biosensor.

The calibration graph drawn between A1AT concentrations and obtained Rct values is given in Figure 9. The measurements obtained by using seven different biosensors were performed for each concentration of A1AT solution, the arithmetic mean of Rct values determined in each measurement were calculated, and the Rct values are given in Figure 9. As shown in Figure 9, the determination range of the proposed biosensor is 100–600 µg/mL and the R2 value is 0.9944.

Limit of detection (LOD) and limit of quantification (LOQ) were determined to be 11.9 µg/mL and 39.6 µg/mL according to IUPAC recommendations [35]. These results showed that the A1AT biosensor constructed in the study can be used with high precision for the quantification and detection of A1AT protein.

The repeatability of the biosensor was investigated in the same laboratory by the same researcher using samples of four different concentrations with 6 repeated measurements. The average value, standard deviation, and coefficient of variation were calculated. The results are given in Table 2. As can be seen from Table 2, the proposed biosensor has good repeatability. In addition, we evaluated the reproducibility of the proposed biosensor. Reproducibility is one of the most important parameters for biosensors. For this purpose, reproducibility experiments were carried out using 9 different biosensors prepared under optimum immobilization conditions. The regression coefficient and equations of the calibration graphs drawn between Rct values and A1AT concentrations are given in Table 3. As seen in Table 3, the regression coefficients and equations of the biosensors fabricated for A1AT determination were approximate to each other. Hence, the proposed biosensors can be utilized reproducibly for A1AT determination.

**Table 2:** Repeatability of the biosensor.

A1AT concentration(µg/mL)	The average value(µg/mL)	Standard deviation (±)	Coefficient of variation
200	198.4055	5.334447	2.69
300	299.3926	4.002498	1.34
400	397.2775	3.783406	0.95
500	498.3976	8.479354	1.70

**Table 3:** Reproducibility of the biosensor.

Biosensor number	R2	Equation	Dynamic range (µg/mL)
1	0.9914	y = 1.3940x – 40.973	100–600 µg/mL
2	0.9957	y = 1.6619x – 38.58	100–600 µg/mL
3	0.9868	y = 1.4835x – 87.32	100–600 µg/mL
4	0.9789	y = 1.3958x – 114.63	100–600 µg/mL
5	0.9953	y = 1.2886x + 179.8	100–600 µg/mL
6	0.9987	y = 1.8750x – 67.44	100–600 µg/mL
7	0.9955	y = 1.1161x – 45.28	100–600 µg/mL
8	0.9931	y = 1.4295x - 105.77	100–600 µg/mL
9	0.9971	y = 1.4014x + 24.7	100–600 µg/mL

For the determination of the interferation effect of proteins, glucose, and salts present in the serum composition on the responses of the A1AT biosensor, measurements were carried out using six different A1AT samples prepared in artificial serum samples. The obtained results are given in Table 4. As shown in Table 4, the recommended biosensor can be used with high precision for A1AT analysis in serum, as it is unaffected by salts, glucose, and proteins.

**Table 4:** The analysis results of A1AT in artificial serum samples.

A1AT concentration ofartificial serum (µg/mL)	Measured A1ATconcentration (µg/mL)	Recovery (%)
100	118.9 ± 5.11	118.9
200	190.01 ± 3.82	95.0
300	295.86 ± 2.54	98.6
400	381.91 ± 3.14	95.5
500	493.83 ± 3.89	98.8
600	619.46 ± 5.58	103.2

For the detection of A1AT, although a method based on surface plasmon resonance (SPR) was developed in 2015 by Kim and Lee, an electrochemical-based biosensor was first designed by Zhu and Lee in 2017 [10,15]. The first of these biosensors based on SPR is a sandwich-type antibody–aptamer biosensor. A DNA aptamer that is specific to A1AT was attached covalently by crosslinking on gold SPR chips via SAM formation of an 11-mercaptoundecanoic acid monolayer. The binding measurements of A1AT to constructed chips were taken in both buffer and spiked human serum with a subsequent anti-A1AT binding step for further SPR signal amplification [10].

For the electrochemical differential pulse voltammetry (DPV) based biosensor, the electrochemical response of 4-aminophenol (AP) produced enzymatically from 4-amino phenyl phosphate (APP) was then used for the quantitative analysis of A1AT. In the sandwich-type assay, the silver nanoparticles (Ag NPs) could enrich ALP-A1AT antibody amount as carriers, and the carbon nanotubes (CNTs) provide high electrical conductivity and large theoretical surface areas, thus further improving the sensitivity for detecting A1AT. By means of CNTs’ ability to offer high surface area and good electrical conductivity and Ag NPs’ ability to increase the amount of ALP on the sensing surface, ALP could dephosphorylate 4-amino phenyl phosphate (APP) enzymatically to produce electroactive species 4-aminophenol (AP) [15]. The A1AT biosensor developed by Zhu and Lee has high selectivity, repeatability, and stability. Despite their advantages, the complexity in the design of the biosensor leads to high chemical requirements and increases the cost of the prepared biosensor. The excessive chemical diversity preferred during the preparation of the biosensor may be a problem in the optimization processes of the biosensor. Therefore, a new A1AT biosensor design with less chemical diversity, based on only immune recognition of the A1AT antibody, may be beneficial.

The comparison of the proposed biosensor with other A1AT biosensors and ELISA test kits is given in Table 5 Biocompare (2020). Alpha-1-antitrypsin ELISA Kits [online]. u1db7 [accessed 01 September 2020].. As seen in Table 5, the recommended biosensor detection range is 1.9–11.5 µM. Since A1AT levels below 50 mg/dL (9.6 µM) are associated with high risk for AATD, this biosensor can be successfully used to detect A1AT deficiency. It is a clear advantage that the proposed biosensor is fabricated with a simpler chemical strategy than other methods. 

**Table 5:** Comparison of the proposed biosensor and other A1AT biosensors.

Methods	Measurement principle	Dynamic range	LOD	References
Aptamer-based sandwich assay	SPR	10–100 fM	-	(10)
Ag NPs-CNTs-based sandwich assay	DPV	0.05–20 pM	0.01 pM	(15)
Human Alpha-1-AntitrypsinELISA Kit (DLdevelop®)	Sandwich ELISA	31.2–2000 ng/mL	11.37 ng/mL(Sensitivity)	*
Bovine Alpha-1-antitrypsinELISA kit (CUSABIO®)	Colorimetric	78.125–5000 ng/ml.	19.5 ng/ml(Sensitivity)	*
The proposed biosensor	EIS	100–600 µg/mL	11.88 µg/mL	This study

* Biocompare (2020). Alpha-1-antitrypsin ELISA Kits [online]. https://www.biocompare.com/pfu/110627/soids/36440/ELISA_Kit/alpha-1-antitrypsin [accessed 01 September 2020].

## 4. Conclusion

A1AT is one of the most important acute phase proteins, and its level in serum quickly increases in the development of inflammation, while its serum level decrease in individuals with the hereditary disorder AATD. AATD is associated with the development of chronic obstructive pulmonary disease, liver disease, and in rare cases skin panniculitis. The simple A1AT biosensor based on EIS proposed in this study can determine A1AT concentration specifically, inexpensively, and quickly. The required time period for the detection of A1AT is 3 min. Nowadays, immune nephelometry and ELISA methods are used to determine A1AT levels in serum. However, these have some analytical disadvantages such as time, cost, and requirement for equipment and a specialist. The proposed biosensor may be a good alternative to this method based on immunoassay. This biosensor has a dynamic range (100–600 µg/mL), LOD (11.88 µg/mL), and LOQ (39.6 µg/mL) values; it can be successfully used to detect AATD. Furthermore, the aromatic thiol 4MPA was used in the design of an EIS-based biosensor in this study for the first time in the literature. In addition, it is an important advantage that the biosensor design requires very little chemical diversity, so the biosensor can be easily fabricated. In this context, an A1AT biosensor with these characteristics may come to the fore as an analytical tool for AATD diagnosis and monitoring its treatment process.
